# Four‐year HbA1c and LDL‐cholesterol trajectories among individuals with mental disorders and newly developed type 2 diabetes

**DOI:** 10.1002/brb3.2372

**Published:** 2021-09-20

**Authors:** Frederik P. Kristensen, Christopher Rohde, Søren D. Østergaard, Reimar W. Thomsen

**Affiliations:** ^1^ Department of Clinical Epidemiology Department of Clinical Medicine Aarhus University Hospital Aarhus Denmark; ^2^ Department of Affective Disorders Aarhus University Hospital—Psychiatry Aarhus Denmark; ^3^ Department of Clinical Medicine Aarhus University Aarhus Denmark

**Keywords:** cholesterol, diabetes mellitus, glycated hemoglobin a, glycemic control, LDL, mental disorders

## Abstract

The impact of different types of mental disorders on long‐term glycemic and lipid trajectories following newly diagnosed type 2 diabetes (T2D) remains unknown. We used real‐world clinical data in a population‐based cohort to fill this knowledge gap. We found that individuals with new T2D and preexisting personality, anxiety, unipolar depression, or psychotic disorder had higher mean HbA1c levels over 4 years following the onset of T2D, whereas no differences were found regarding LDL‐C levels. This knowledge should be considered in the management of T2D in these vulnerable groups.

## Graphical abstract

We found that individuals with new type 2 diabetes and preexisting personality, anxiety, unipolar depression, or psychotic disorder had higher mean HbA1c levels over 4 years following the onset of T2D, whereas no differences were found regarding LDL‐C levels.

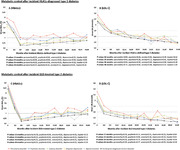

Good glycemic and lipid control is critical to prevent development of disabling micro and macrovascular complications in type 2 diabetes (T2D) (ADA: Standards of Medical Care in Diabetes‐2021, 2021). Results of a recent study suggested that individuals with new T2D and preexisting depression are as likely to successfully attain early Hemoglobin A1C (HbA1c) and low‐density lipoprotein cholesterol (LDL‐C) targets as are T2D individuals without depression (Rohde et al., 2021). However, the impact of depression and other mental disorders on long‐term glycemic and lipid trajectories remain unknown. We used data from unique population‐based Danish registers to fill this knowledge gap (Arendt et al., 2020; Schmidt et al., 2019).

We identified two nonmutually exclusive populations with incident T2D among residents of the Central and Northern Denmark Regions in the period from 2000 to 2016:

1. Incident HbA1c‐defined T2D: Individuals with their first measurement of elevated HbA1c (≥6.5% [48 mmol/mol]) at age ≥30 years.
2. Incident glucose lowering drug (GLD)‐treated T2D: Individuals that redeemed their first prescription for a GLD at age ≥30 years.

For both cohorts, we excluded individuals with a prior redeemed prescription for a GLD or prior hospital‐based diagnosis of diabetes [Anatomical Therapeutic Chemical (ATC) code A10 and International Classification of Diseases (ICD‐10) codes E10‐E15, E160‐E162, O24, T383A, M142, G590, G632, H280, H334, H450, H360, or N083]. Within these two cohorts, we identified all individuals who had been diagnosed (not mutually exclusive) with psychotic disorder (ICD‐10 codes: F20‐F29), bipolar disorder (ICD‐10 codes: F30‐F31), unipolar depression disorder (ICD‐10 codes: F32‐F33), anxiety disorder (ICD‐10 codes: F40‐F49), or personality disorder (ICD‐10 codes: F60‐F61) in the 3 years preceding the diabetes index date. We included all diagnoses in relation to inpatient, outpatient, or emergency room treatment at any psychiatric hospital in Denmark. For both cohorts, we extracted data on all available HbA1c and LDL‐C values from the diabetes diagnosis date and until death, emigration, or 4 years postindex date, whichever came first. The HbA1c and LDL‐C values were pooled across 4‐month intervals and comparisons of mean levels between cohort members with and without mental disorders were performed using the two‐sample *t*‐test.

Figure 1 shows the trajectories of mean HbA1c and LDL‐C levels, stratified by mental disorder status. Among those with incident HbA1c‐defined T2D, the mean HbA1c levels at baseline were between 7.5% (58 mmol/mol) and 8.0% (64 mmol/mol), and then decreased by approximately 1% point over the course of approximately eight months, to mean levels between 6.6% (49 mmol/mol) and 7.0% (53 mmol/mol) (Figure 1a). Subsequently, a slight gradual increase in HbA1c levels were observed for all subgroups, but the mean HbA1c levels generally remained under the recommended 7% (53 mmol/mol) target (ADA: Standards of Medical Care in Diabetes‐2021, 2021), except for the cohort members with personality, anxiety, or psychotic disorder. Specifically, individuals with personality, anxiety, unipolar depression, or psychotic disorder had higher (0.10%–0.36%) mean HbA1c levels compared to individuals without mental disorder, both at baseline and throughout follow‐up (see Figure 1a for *p* values). The mean LDL‐C levels at baseline were between 3.05 mmol/L and 3.34 mmol/L across groups (Figure 1b). During follow‐up, the mean LDL‐C gradually decreased in all subgroups to levels below the recommended 2.6 mmol/L (ADA: Standards of Medical Care in Diabetes‐2021, 2021) at 48 months. Although the mean LDL‐C levels tended to be higher for individuals with versus without mental disorders, no statistically significant differences were found (Figure 1b).

**FIGURE 1:**
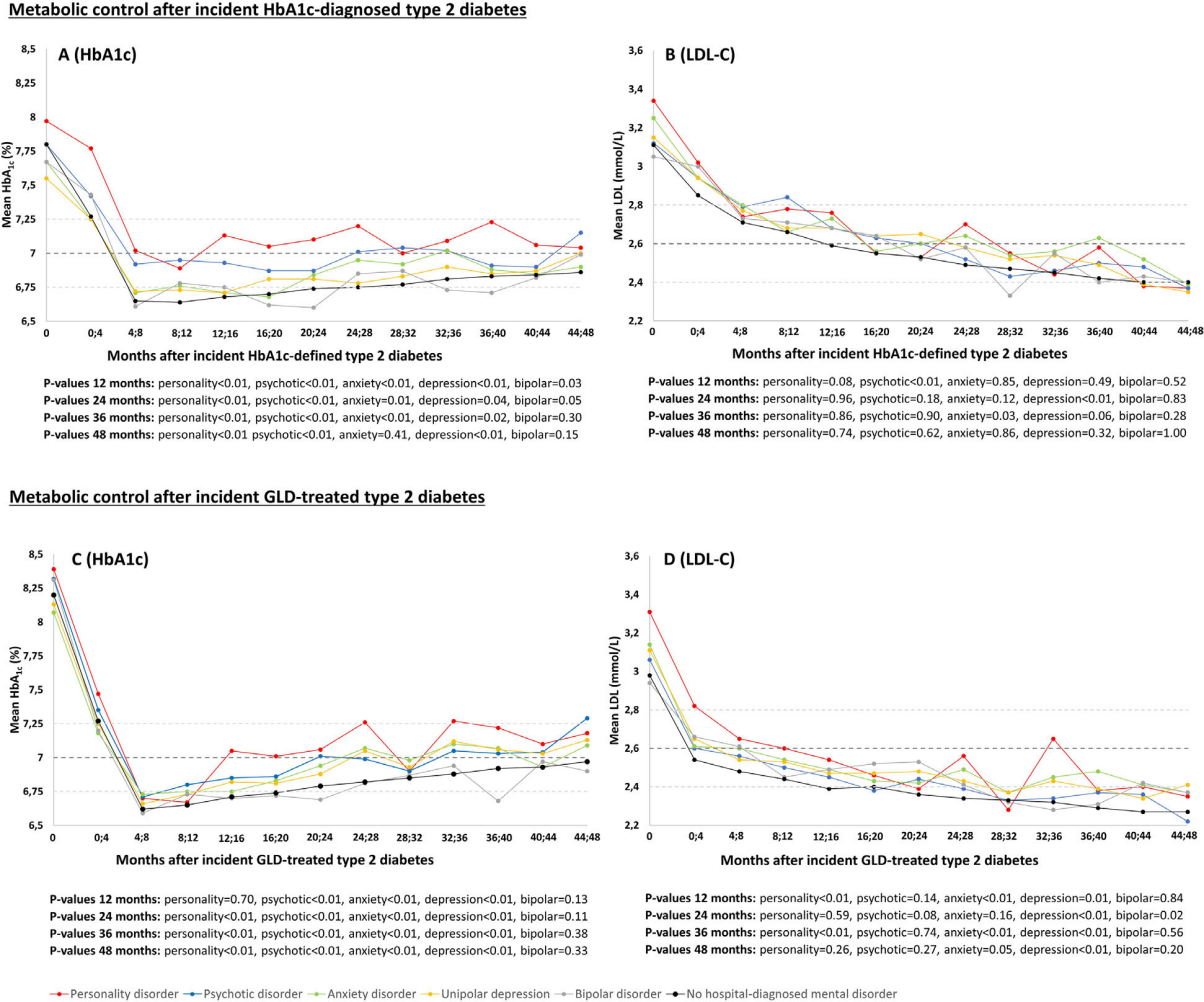
Trajectories of mean HbA1c and mean LDL‐C levels based on everyday clinical practice measurements following incident HbA1c‐defined (a, b) or incident GLD‐treated (c, d) T2D, stratified according to history of hospital‐diagnosed mental disorders. HbA1c and LDL‐C: HbA1c and LDL‐C values from measurements ordered by any healthcare provider at any patient visit in the primary or secondary health care sector were included. The mean HbA1c and LDL‐C levels were calculated for intervals spanning 4 months, using all available HbA1c and LDL‐C values within these intervals. *p* values: We used a *t*‐test to compare HbA1c and LDL‐C levels measured at 12, 24, 36, and 48 months of follow‐up in individuals with a mental disorder versus no mental disorder. Number of individuals with incident HbA1c‐defined T2D: Personality disorder: 392, psychotic disorder: 753, anxiety disorder: 966, unipolar depression disorder: 1260, bipolar disorder: 284, no hospital‐diagnosed mental disorder: 85,835. Proportion initiating GLDs among individuals with incident HbA1c‐defined T2D diabetes (median time to GLD, quartiles): 77.3% with personality disorder (67 days, 12–505), 72.6% with psychotic disorder (124 days, 14–522), 71.3% with anxiety disorder (75 days, 13–487), 66.1% with unipolar depression disorder (84 days, 13–540), 65.5% with bipolar disorder (99 days, 15–550), and 67.4% with no mental disorder (112 days, 11–792). Proportion initiating or that are treated with a lipid‐modifying agent in the year leading up to incident HbA1c‐defined T2D diabetes: 73.1% with anxiety disorder, 68.7% with bipolar disorder, 68.7% with unipolar depression disorder, 68.6% with personality disorder, 65.1% with psychotic disorder, and 72.0% with no mental disorder. Number of individuals with incident GLD‐defined T2D diabetes: Personality disorder: 559, psychotic disorder: 926, anxiety disorder: 1240, unipolar depression disorder: 1520, bipolar disorder: 381, no hospital diagnosed mental disorder: 101,472

Among those with incident GLD‐defined T2D, baseline HbA1c levels were higher than for the HbA1c‐defined diabetes cohort (as expected since decision of GLD initiation is a proxy for severity). However, the HbA1c and LDL‐C trajectories over time were similar to those described above for the HbA1c‐defined cohort (Figure 1c, d).

In this study of incident T2D, we report that, compared to individuals without mental disorders, those with personality, anxiety, unipolar depression, or psychotic disorder had higher mean HbA1c levels over 4 years following the onset of T2D. No differences were found regarding LDL‐C levels. These findings suggest that the management of T2D in individuals with personality, anxiety, unipolar depression, or psychotic disorder is suboptimal, possibly due to challenges with self‐care or lacking initiation of/compliance with GLD treatment and control regimens (Ward & Druss, 2015).

A central limitation to this study is that prior to 2012, HbA1c measurement was restricted mainly to individuals with anticipated problems regarding glycemic control and rarely used diagnostically. This may have caused selective inclusion of high HbA1c levels before 2012. Furthermore, only ≈50% of the patients had an HbA1c measurement taken in each 4‐month interval. This proportion was however independent of mental disorder status (data not shown), which speaks against bias in the comparisons.

In conclusion, compared to those without mental disorder, individuals with T2D and personality, anxiety, unipolar depression, or psychotic disorder had higher HbA1c levels over time. This knowledge should be considered in the management of T2D in these vulnerable groups.

## DECLARATION OF INTEREST AND FUNDING

CR is supported by the Danish Diabetes Academy, funded by the Novo Nordisk Foundation (grant number NNF17SA0031406), the International Diabetic Neuropathy Consortium (IDNC), which is supported by a Novo Nordisk Foundation Challenge Programme grant (grant number NNF14OC0011633), and the Lundbeck Foundation(grant number R358‐2020‐2342). SDØ is supported by the Novo Nordisk Foundation (grant number: NNF20SA0062874), the Lundbeck Foundation (grant numbers: R358‐2020‐2341 and R344‐2020‐1073), The Danish Cancer Society (R283‐A16461), and Independent Research Fund Denmark (grant number: 7016‐00048B). The Department of Clinical Epidemiology is involved in studies with funding from various companies as research grants to (and administered by) Aarhus University. None of these studies have relation to the present study. No other potential conflicts of interest relevant to this article were reported.

## AUTHOR CONTRIBUTIONS

All authors contributed to the design of the study and the interpretation of the data. CR conducted the statistical analyses. FPK and CR drafted the first version of the manuscript. SDØ and RWT revised the manuscript for important intellectual content. All authors approved the final version of the manuscript. CR and RWT are the guarantors of this work and, as such, had full access to all the data in the study and take responsibility for the integrity of the data and the accuracy of the data analysis.

### PEER REVIEW

The peer review history for this article is available at https://publons.com/publon/10.1002/brb3.2372

## Data Availability

No additional data available.

## References

[brb32372-bib-0001] (2021). ADA: Standards of medical care in diabetes‐2021. Diabetes Care, 44(Suppl 1), S1–S232.33298409

[brb32372-bib-0002] Arendt, J. F. H. , Hansen, A. T. , Ladefoged, S. A. , Sørensen, H. T. , Pedersen, L. , & Adelborg, K. (2020). Existing data sources in clinical epidemiology: Laboratory information system databases in Denmark. Clinical Epidemiology, 12, 469–475. 10.2147/CLEP.S245060 32547238 PMC7244445

[brb32372-bib-0003] Rohde, C. , Knudsen, J. S. , Schmitz, N. , Østergaard, S. D. , & Thomsen, R. W. (2021). The impact of hospital‐diagnosed depression or use of antidepressants on treatment initiation, adherence and HbA(1c)/LDL target achievement in newly diagnosed type 2 diabetes. Diabetologia, 64(2), 361–374. 10.1007/s00125-020-05303-4 33073329

[brb32372-bib-0004] Schmidt, M. , Schmidt, S. A. J. , Adelborg, K. , Sundboll, J. , Laugesen, K. , Ehrenstein, V. , & Sorensen, H. T. (2019). The Danish health care system and epidemiological research: From health care contacts to database records. Clinical Epidemiology, 11, 563–591. 10.2147/CLEP.S179083 31372058 PMC6634267

[brb32372-bib-0005] Ward, M. , & Druss, B. (2015). The epidemiology of diabetes in psychotic disorders. Lancet Psychiatry, 2(5), 431–451. 10.1016/S2215-0366(15)00007-3 26360287

